# Nomenclature for renal replacement therapy and blood purification techniques in critically ill patients: practical applications

**DOI:** 10.1186/s13054-016-1456-5

**Published:** 2016-10-10

**Authors:** Gianluca Villa, Mauro Neri, Rinaldo Bellomo, Jorge Cerda, A. Raffaele De Gaudio, Silvia De Rosa, Francesco Garzotto, Patrick M. Honore, John Kellum, Anna Lorenzin, Didier Payen, Zaccaria Ricci, Sara Samoni, Jean-Louis Vincent, Julia Wendon, Marta Zaccaria, Claudio Ronco, on behalf of the Nomenclature Standardization Initiative (NSI) Alliance, S. M. Bagshaw, A. Balducci, I. Baldwin, F. Barbarigo, R. Bellomo, P. Braganò, J. Braunsky, F. Calletti, J. Cerda, L. Chawla, S. De Rosa, S. Di Somma, K. Doi, P. Dosil Rosende, M. Emmett, L. Fecondini, D. Galavotti, F. Garzotto, N. Gibney, S. L. Goldstein, G. Guadagni, P. Honoré, E. Hoste, S. Inadome, K. Kashani, N. Katz, J. Kellum, R. Kenley, Y. Kobayashi, J. Lannoy, A. Lewington, A. Lorenzin, F. Mariano, P. A. McCullough, R. L. Mehta, J. Menneguerre, M. Mettifogo, M. Neri, M. Ostermann, A. Pani, P. Pirazzoli, R. Pohlmeier, D. Pouchoulin, Z. Ricci, C. Ronco, M. Rosner, M. Seamann, A. Shaw, A. Tolwani, G. Villa, J. L. Vincent, J. Wendon

**Affiliations:** 1 Department of Nephrology, Dialysis and Transplantation, International Renal Research Institute of Vicenza, San Bortolo Hospital, Viale Rodolfi 37, 36100 Vicenza, Italy; 2 Department of Health Sciences, Section of Anaesthesiology, Intensive Care and Pain, University of Florence, Florence, Italy; 3 Department of Management and Engineering, Universityof Padova, Vicenza, Italy; 4 Department of Intensive Care, Austin Hospital, Department of Epidemiology and Preventive Medicine, Australian and New Zealand Intensive Care Research Centre, Monash University, Melbourne, Victoria Australia; 5 Department of Medicine, Albany Medical College, Albany, New York 12209 USA; 6 Intensive Care Department, Universitair Ziekenhuis Brussel, Vrije Universiteit Brussel, Brussels, Belgium; 7 Center for Critical Care Nephrology, Department of Critical Care Medicine, University of Pittsburgh, Pittsburgh, PA USA; 8 Service d’Anesthésie-Réanimation-SMUR, Lariboisière AP-HParis, Université Paris Diderot-Paris, Paris, France; 9 Department of Pediatric Cardiac Surgery, Bambino Gesù Children’s Hospital, Rome, Italy; 10 Institute of Life Sciences, Sant’Anna School of Advances Studies, Pisa, Italy; 11 Department of Intensive Care, Erasme Hospital, Université libre de Bruxelles, Brussels, Belgium; 12 Liver Intensive Therapy Unit, Institute of Liver Studies, King’s College London, Denmark Hill Campus, London, UK

**Keywords:** Terminology, Pump, Pressure sensor, CRRT machine, Continuous veno-venous hemodialysis, Continuous veno-venous hemofiltration, Continuous veno-venous hemodiafiltration, High volume hemofiltration, Continuous plasmafiltration coupled with adsorption, Hemoperfusion

## Abstract

This article reports the conclusions of the second part of a consensus expert conference on the nomenclature of renal replacement therapy (RRT) techniques currently utilized to manage acute kidney injury and other organ dysfunction syndromes in critically ill patients. A multidisciplinary approach was taken to achieve harmonization of definitions, components, techniques, and operations of the extracorporeal therapies. The article describes the RRT techniques in detail with the relevant technology, procedures, and phases of treatment and key aspects of volume management/fluid balance in critically ill patients. In addition, the article describes recent developments in other extracorporeal therapies, including therapeutic plasma exchange, multiple organ support therapy, liver support, lung support, and blood purification in sepsis. This is a consensus report on nomenclature harmonization in extracorporeal blood purification therapies, such as hemofiltration, plasma exchange, multiple organ support therapies, and blood purification in sepsis.

## Background

The use of renal replacement therapy (RRT) in the management of acute kidney injury (AKI) requires a multidisciplinary approach. It is, therefore, essential that all members of the team use the same terminology, but the terms used to describe the different modalities of RRT often vary and can be confusing. In this article, we provide an updated consensus nomenclature to help navigate this complex field. We review the practical applications of transmembrane solute and fluid transport principles and the control mechanisms for RRT devices. The article focuses on continuous renal replacement therapies (CRRTs), which are commonly used in the treatment of critically ill patients. We hope that this standardized terminology will be adopted by all involved in this field, including industry as they develop new devices.

## Methodology

A conference was organized in Vicenza, Italy, to gather experts in CRRT and members of companies manufacturing CRRT hardware and devices to establish consensus on technical terminology and definitions relevant to basic principles of CRRT and related technologies [1]. The conference provided the background for a modified Delphi consensus methodology as previously utilized for the Acute Disease Quality initiative consensus sessions [2]. Prior to the conference, participants screened the literature of the last 25 years and previous taxonomy efforts [3–5]. Keywords included “continuous renal replacement therapy”, “dialysis”, “hemofiltration”, “convection”, “diffusion”, “ultrafiltration”, “dose”, “blood purification”, “renal support”, “multiorgan dysfunction”, together with the relative MeSH (Medical Subject Headings) terms. Abstracts of 707 articles were screened and more than 300 papers were read in full and analyzed. Based on this literature search, a series of definitions and terms were proposed and consensus was achieved from the majority of experts who participated in the conference. Where consensus was lacking, different statements were created after two-thirds of the audience expressed a positive vote. We present the results of this effort of terminology harmonization called the Nomenclature Standardization Initiative (NSI).

## Hardware and devices

CRRT “hardware” includes the machine and all dedicated disposables. Knowledge of the nomenclature and the functions of the machine and its main components is extremely important, not only for nurses and technicians but also for clinicians.

**Fig. 1:**
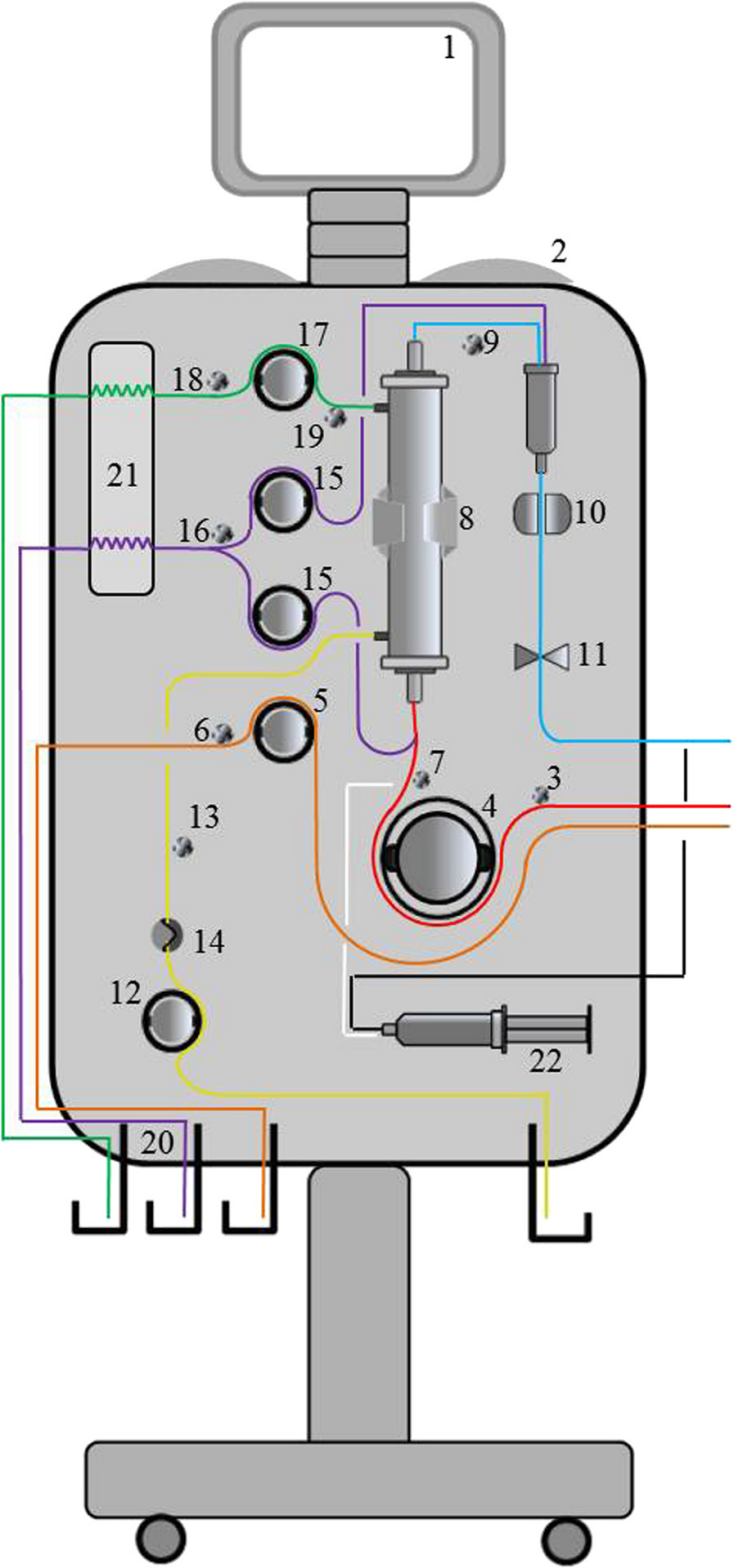
The CRRT machine (see the text for explanation of numbered components)

Figure 1 depicts a standard CRRT machine equipped with current technology and characteristics [6, 7]. Its main components include:

1. *Screen*: the monitor through which the user interacts with the machine.
2. *Alarm light and sound indicators*: visual and auditory alarms must be clear and comprehensive. The alarm settings should be unequivocally categorized according to a specific standard.
3. *In-flow pressure (P*_*IN*_ *) sensor* (upstream of blood pump): monitors the negative pressure in the blood in-flow line between the patient’s vascular access and the blood pump.
4. *Blood pump*: pump that controls the blood flow rate through the extracorporeal circuit.
5. *Pre-blood pump*: pump that controls the flow rate of solutions, mainly regional anticoagulants (e.g., citrate), into the blood in-flow line before the blood pump.
6. *Pre-blood pump pressure sensor*: monitors the pressure before the pre-blood pump.
7. *Pre-filter pressure (P*_*PRE*_ *) sensor* (downstream of blood pump): located in the blood flow line between the blood pump and filter, this sensor monitors the positive pressure and enables calculation of the transmembrane pressure (TMP) and pressure drop (P_DROP_) in the filter.
8. *Filter holder*: holds the filter or the entire filter-tubing kit on the machine.
9. *Out-flow pressure (P*_*OUT*_ *) sensor*: monitors the positive pressure between the filter and the patient vascular access. This sensor is used to calculate the TMP and pressure drop in the filter.
10. *Bubble detector*: a transducer that detects the presence of air in the blood out-flow line.
11. *Safety out-flow electroclamp*: a mechanism that produces occlusion of the blood out-flow line.
12. *Effluent/ultrafiltrate pump*: pump that controls the rate of total fluid removal from the filter.
13. *Effluent/ultrafiltrate pressure (P*_*EFF*_ *) sensor*: monitors the pressure in the effluent compartment of the filter. This sensor is placed before the effluent pump and allows calculation of the TMP.
14. *Blood leak detector (BLD)*: placed along the effluent line, it identifies unwanted blood leaks from the blood compartment of the filter.
15. *Replacement/infusion pump*: the pump that controls the rate of replacement fluid flow into the blood in-flow line (pre-dilution, usually between the blood pump and the filter) and/or into the blood out-flow line (post-dilution, usually in the blood out-flow chamber, such as the deaeration or venous drip chamber).
16. *Pre-replacement pump pressure sensor*: monitors the negative pressure before the replacement pump.
17. *Dialysate pump*: the pump that controls the rate of dialysate flow into the filter.
18. *Pre-dialysate pump pressure sensor*: monitors the negative pressure before the dialysate pump.
19. *Post-dialysate pump pressure (P*_*Di*_ *) sensor*: monitors the pressure in the dialysate line before the connection with the filter. Permits a better estimate of TMP.
20. *Fluid control system*: allows direct monitoring of the fluid balance related to fluids exchanged by the CRRT machine during the treatment. It can be gravimetric, volumetric, fluximetric, or a combination of these mechanisms.
21. *Heater*: heats the dialysate/replacement fluids or the blood flowing through the blood out-flow line of the extracorporeal circuit.
22. *Anticoagulant pump*: infuses anticoagulants into the blood circuit. Depending on the anticoagulation modality chosen, this pump can be a single unit (systemic anticoagulation, e.g., heparin) or be a part of a more complex infusion system with multiple pumps. In fact, in cases of regional anticoagulation (heparin-protamin or citrate-calcium) a second pump is necessary to infuse the antagonist of the selected anticoagulant into the blood out-flow line.

## CRRT machine: procedures and phases of treatment

The different procedures performed by the machine [8] include:

- *Prescription phase*: this phase consists of decisions by the prescribing clinician about the required modality and related operational parameters and includes periodic reassessment and/or change of the prescription.
- *Preparation phase*: this phase consists of collection of necessary disposable material, identification and checking of the disposable set, set loading (cassette tubing), connection to the filter, positioning of the tubing, and hanging of bags.
- *Priming phase*: priming solution is infused into the extracorporeal circuit in order to remove air and impurities remaining after sterilization of the set. When heparin anticoagulation is used, it is usually added to the priming solution. During this phase, the machine makes a general check of all components and sensors.
- *Connection to the patient*: this phase consists of the connection of the extracorporeal lines to the patient’s vascular access.
- *Treatment phase*: net ultrafiltration and diffusive and/or convective solute transport are activated (all the pumps are working) and blood purification is performed. The patient’s vital signs and circuit pressures must be monitored throughout the treatment phase.
- *Special procedures*: during treatment, special procedures can include replenishment of dialysate, replacement fluid, and citrate bags (when citrate anticoagulation is used), change of syringes (when using heparin anticoagulation), repositioning of the vascular access, and temporary disconnection, recirculation, and replacement of filter and kit.
- *Blood return, disconnection and unload*: the blood return procedure returns the blood to the patient. This is usually done by connecting a saline solution bag to the in-flow blood line and running the blood pump. When the circuit is flushed, the blood pump is stopped, the blood outflow line disconnected, and the tubing and filter unloaded.

## CRRT disposables

Disposables (single-use components of the extracorporeal circuit) are specific for every machine and are usually designed for a specific treatment modality. The main disposables [9] and color codes that should mark each tubing line are listed in Table 1.

**Table 1:** Main disposables and their components with associated color code in a CRRT extracorporeal circuit (modified from [45])

**Tubes**	
Blood in-flow line (red; previously known as access or arterial line)	Segment connecting the patient’s vascular access to the filter
	Segment for pressure measurement (upstream blood pump): segment of the blood in-flow line connected to the in-flow pressure sensor
	Pump segment line: segment inserted between the rotor and the stator of the blood pump
	Blood in-flow air removal chamber: allows removal of light air bubbles before the blood enters the filter
	Segment for pressure measurement (downstream blood pump): segment of the blood in-flow line connected to the pre-filter pressure sensor
Blood out-flow line (dark blue; previously known as return or venous line)	Segment connecting the filter to the patient’s vascular access
	Segment for pressure measurement: segment of the blood out-flow line connected to the out-flow pressure sensor
	Blood out-flow air removal chamber: allows removal of light air bubbles before the blood returns to the patient
Effluent/ultrafiltrate line (yellow)	Segment that allows the flow of waste fluids from the filter
	Pump segment line: segment inserted between the rotor and the stator of the effluent/ultrafiltrate pump
	Segment for pressure measurement: segment of the effluent line connected to the effluent/ultrafiltrate pressure sensor
Dialysate line (green)	Segment that allows the flow of incoming dialysate into the filter
	Pump segment line: segment inserted between the rotor and the stator of the dialysate pump
	Segment for pressure measurement (if present): segment of the dialysate line connected to the dialysate pressure sensor
	Heater line: segment of the dialysate line placed in contact with the heater
Replacement line (purple or light blue)	Segment that allows the flow of replacement fluid into the blood in-flow and/or blood out-flow lines
	Pump segment line: segment inserted between the rotor and the stator of the replacement pump
	Segment for pressure measurement (if present): segment of the replacement line connected to the replacement pressure sensor
	Heater line: segment of the replacement line placed in contact with the heater
Pre-blood line (orange)	Segment that allows the flow of specific fluids (mainly regional anticoagulants) into the blood in-flow line before the blood pump
	Pump segment line: segment inserted between the rotor and the stator of the pre-blood pump
	Segment for pressure measurement (if present): segment of the pre-blood line connected to the pre-blood pressure sensor
Anticoagulant and specific antagonists line	Segments connecting the anticoagulant/specific antagonist bag or pump to the main blood circuit
	Citrate line (orange): segment for citrate infusion (i.e., pre-blood line)
	Heparin line (white): segment connecting the heparin syringe pump to the blood in-flow line
	Specific antagonist line (black): segment connecting the specific antagonist syringe pump to the blood out-flow line
**Filter**	
Fiber (membranes)	Every fiber, hollow and of cylindrical shape, allows the transport of fluids and solutes through their porous semi-permeable surface
Bundle	Entire number of fibers inside the housing
Housing	Plastic casing containing a single membrane fiber bundle
	Blood in-flow port: entrance port of blood entering into the filter
	Blood out-flow port: exit port of blood leaving the filter
	Dialysate in-flow port: entrance port of fresh dialysate
	Effluent/ultrafiltrate out-flow port: exit port of waste solution
Potting	Polyurethane component fixing the bundle within the housing and embedding the bundle at both ends of the filter

During CRRT, the filter is the key disposable through which blood or plasma is effectively purified by ultrafiltration, convection, and/or diffusion. Historically, the designation “filter” describes the entire purifying extracorporeal device system (i.e., membranes, housing, etc.). Among the different types of filters, hemofilters, hemodialyzers, and hemodiafilters should be used when exclusively convective, diffusive, or convective plus diffusive modalities, respectively, are applied. In this article we use these terms distinctly, taking into account the different CRRT modalities. A plasma filter is defined as a specific filter that allows the separation of plasma from cellular elements. Sorbents, cartridges, and adsorbers do not belong to the category of filters; in this case, adsorption is the only purifying modality. The only available type of CRRT filter that can perform diffusive and/or convective transport is shaped as a collection of parallel “hollow fibers”. The filters can be mainly identified by membrane geometrics and performance characteristics [10–12].

## Volume management and fluid balance

Fluid management during CRRT must take into account the volume and hemodynamic status of the patient. The machine fluid balance error is the fluid management error caused by CRRT machine malfunction. Based on the inherent variability (“tolerances”) in the performance of the fluid pumps, scales, and other components of a CRRT machine’s fluid management system, the manufacturer provides a specified limit (“specification”) beyond which a fluid imbalance is considered an error. Fluid imbalances can be due to hardware (scales, pumps, tubes) or software (control system and protective subsystem) errors. Various systems have been proposed for fluid balancing in CRRT machines:

- Gravimetric fluid balancing, using one or more scales, is most commonly used in CRRT because it is the most reliable technique during long treatment intervals. A fundamental aspect of this type of system is the continuous weighing of the effluent along with replacement fluid and/or dialysate, with weight acting as a surrogate for fluid flow rate. The machine software analyzes these scale data on an ongoing basis and any discrepancies between prescribed and actual values lead to adjustments in pump rates based on a servo-feedback mechanism. Disadvantages include limitations in scale capacity, user errors, and other disturbances of the operating environment.
- In volumetric fluid balancing, a system of balancing chambers and valves is used. During long treatments, volumetric balancing is less accurate than gravimetric balancing because of systematic, cumulative errors, as there is no continuous servo-feedback safeguard for this approach. The advantage of this system is that it eliminates the need to collect effluent and thus reduces the frequency of fluid-related interventions.
- Fluxometric fluid balancing requires the application of accurate but expensive flow meters (electromagnetic, ultrasonic and Coriolis flow meters).

All these methods can be applied individually or in combination.

## Extracorporeal therapies and treatments

Extracorporeal therapies can be categorized according to session frequency and duration.

### Continuous therapies

**Fig. 2:**
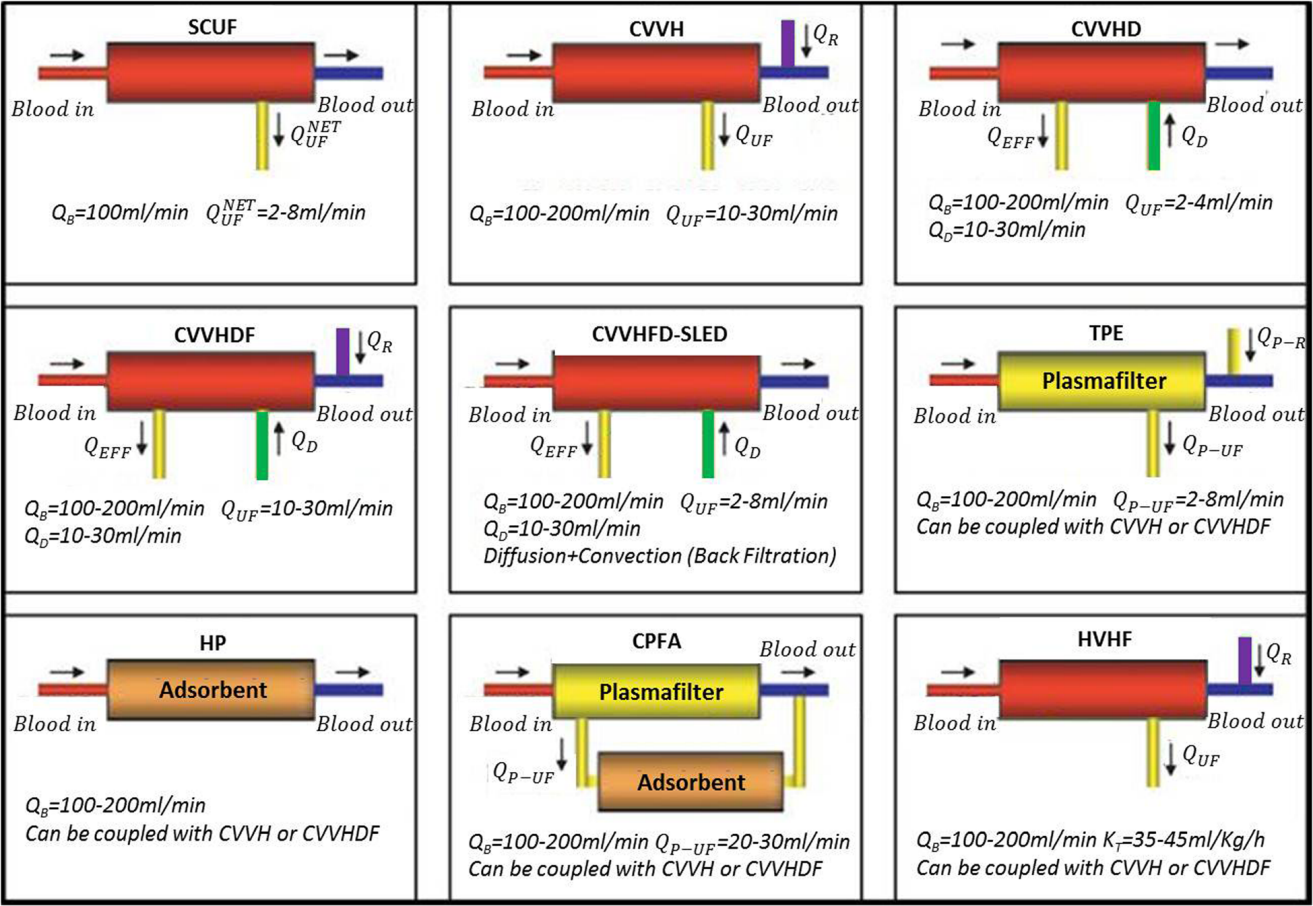
Main extracorporeal therapies and treatments (modified from [5]) *Abbreviations*: *Q*_*B*_ blood flow rate, *Q*_*UF*_^*NET*^ net ultrafiltration flow rate, *Q*_*UF*_ ultrafiltration flow rate, *Q*_*D*_ dialysate flow rate, *Q*_*R*_ total replacement flow rate, *Q*_*EFF*_ effluent flow rate, *Q*_*P-R*_ replacement plasma flow rate, *Q*_*P-UF*_ plasma ultrafiltration flow rate, *SCUF* slow continuous ultrafiltration, *CVVH* continuous veno-venous hemofiltration, *CVVHD* continuous veno-venous hemodialysis, *CVVHDF* continuous veno-venous hemodiafiltration, *CVVHFD-SLED* continuous veno-venous high-flux dialysis–sustained low-efficiency dialysis, *TPE* therapeutic plasma exchange, *HP* hemoperfusion, *CPFA* continuous plasma filtration coupled with adsorption, *HVHF* high-volume hemofiltration

CRRT is any extracorporeal technique that replaces kidney function and more generally provides blood purification for an extended period of time. CRRT is considered by many clinicians to be the most appropriate modality for the management of hemodynamically unstable patients with AKI, promoting better hemodynamic stability, reduced transcellular solute shifts, and better tolerance to fluid removal than intermittent extracorporeal therapies. The need for expertise, the necessity of continuous anticoagulation, the nursing workload, the continuous alarm vigilance, and the higher costs are some of the limitations of this approach. CRRT can be provided in various forms depending on resources, patient needs, and staff skills [5, 13, 14] (Fig. 2).

Prescription should be reviewed regularly.

CRRT treatments are currently performed using a double lumen catheter as vascular access, a “veno-venous” technique whereby blood is driven from a vein and, after being purified, returned to the same vein. “Arterio-venous” circuits have been virtually abandoned.

#### Slow continuous ultrafiltration

Slow continuous ultrafiltration (SCUF), based only on slow removal of plasma water, is used for patients with refractory fluid overload, with or without renal dysfunction. Its primary aim is to achieve safe and effective correction of fluid overload.

#### Continuous veno-venous hemofiltration

Continuous veno-venous hemofiltration (CVVH) uses convection, with ultrafiltrate replaced in part or completely with appropriate replacement fluids, to achieve solute clearance and volume control. Replacement fluid can be infused before (predilution) and/or after (postdilution) the hemofilter.

#### Continuous veno-venous hemodialysis

Continuous veno-venous hemodialysis (CVVHD) is a form of continuous hemodialysis characterized by counter-current/co-current dialysate flow rate into the dialysate compartment of the hemodialyzer. The main mechanism of transmembrane solute transport is diffusion.

#### Continuous veno-venous hemodiafiltration

Continuous veno-venous hemodiafiltration (CVVHDF) combines hemodialysis and hemofiltration modalities. Ultrafiltrate is replaced in part or completely by replacement fluid (pre- or post-infusion) and counter-current/co-current dialysate flow into the dialysate compartment. Solute clearance is achieved via diffusive and convective clearance.

#### Continuous veno-venous high-flux hemodialysis

Continuous veno-venous high-flux hemodialysis (CVVHFD) consists of the same treatment as in CVVHD but carried out using high-flux membranes. Due to the high-flux properties of the membrane, a convective component of solute clearance is achieved even if replacement fluid is not infused.

### Intermittent therapies

Intermittent therapies are carried out in sessions of 3–5 h. They require adequate vascular access, specially trained nurses, and water processing and sterilization that produces pure water for dialysate. Since treatment times are relatively short, the depuration rate must be higher than that of CRRT. The most commonly prescribed intermittent therapies are intermittent hemodialysis (IHD), intermittent hemofiltration (IHF), intermittent hemodiafiltration (IHDF), and intermittent high-flux dialysis (IHFD). Other therapies are available combining different modalities but these are not usually performed in the intensive care unit (ICU), so are not discussed here.

### Hybrid therapies

With respect to frequency and duration, the term “hybrid therapies” relates to the blending of characteristics from both intermittent and continuous modalities. These therapies attempt to optimize the advantages and minimize the disadvantages of both modalities: efficient solute removal, slower ultrafiltration rates for hemodynamic stability, less anticoagulant exposure, shorter duration, lower costs, decreased nurse workload, and improved ICU workflow. Hybrid therapies encompass various specific “discontinuous” RRT modalities: sustained low-efficiency dialysis (SLED), slow low-efficiency extended daily dialysis (SLEDD), prolonged intermittent RRT (PIRRT), extended daily dialysis (EDD), extended daily dialysis with filtration (EDDf), extended dialysis (ED), “go slow dialysis”, and accelerated veno-venous hemofiltration (AVVH).

Hybrid therapies are usually performed with standard intermittent hemodialysis equipment, including machines, filters, extracorporeal blood circuits, and, in some cases, online fluid production for dialysate and ultrafiltrate infusion. Solute removal is largely diffusive but variants with a convective component, such as EDDf and AVVH, are possible.

The most commonly prescribed hybrid therapy is SLED, a technique that uses reduced blood and dialysate flow rates and is usually limited to 8–12 h. Data from appropriately powered studies on the application of these techniques are limited [15].

### Other extracorporeal therapies

Other blood purification techniques are also performed in the ICU to clear toxins and solutes generally not removable by “classic” RRT or to support single or multiple organ dysfunction. While the delivery of CRRT may be achieved without anticoagulation in some patients, these therapies typically require some form of anticoagulation.

#### Therapeutic plasma exchange

Therapeutic plasma exchange (TPE) consists of the automated removal of plasma (plasmapheresis) and its replacement (exchange) with a suitable fluid composed of fresh frozen plasma or albumin.

TPE is performed using a centrifugal-based system or a very highly permeable membrane that allows separation of plasma from the cellular elements of blood. In membrane-based TPE, pore sizes ranging between 0.2 and 0.6 microns allow a sieving coefficient of 0.9–1.0 for molecules with a molecular weight greater than 500 kDa [16].

Continuous plasma exchange (CPE) is a therapy derived from TPE that is performed with lower flow rates and for a longer period of time. Single or repeated sessions can be performed as pure CPE or in conjunction with other purification techniques.

#### Multiple organ support therapy

Recently, CRRT has been used in a wide range of “non-renal applications”, including multiple organ support therapies (MOSTs), to manage patients with multiple organ dysfunction syndrome [17]. MOST requires a complex extracorporeal support system with a multi-tasking machine platform and multiple devices. The type and intensity of organ support therapy can be modulated according to the number and severity of organ dysfunctions.

##### Heart support

In myocardial dysfunction, right and left ventricular dysfunction can be complicated by severe fluid overload [18]. SCUF, performed in patients with or without AKI, can reduce fluid overload, improve cardiac filling volumes and contractility and is usually well tolerated among hemodynamically unstable cardiac failure patients [19]. It may be especially worthy of consideration in patients with severe diuretic resistance and cardiorenal syndrome, for whom therapeutic options are limited.

##### Liver support

Artificial liver support includes “cell-based” and “non-cell-based” devices, including conventional IHD, CRRT, and devices specifically designed to clear accumulated toxins associated with liver dysfunction [20, 21] (Table 2). In many non-cell-based systems, an albumin-enriched dialysate is necessary to remove such toxins (e.g., fatty acids, hydrophobic bile acids, and nitric oxide), which are highly albumin-bound. This “albumin dialysis” concept forms the basis of single pass albumin dialysis (SPAD) and the molecular adsorbent recirculating system (MARS) while Prometheus (Fresenius Medical Care, Bad Homburg, Germany) is based on fractionated plasma separation and adsorption (FPSA) [22].

**Table 2:** Liver support systems in the hepato-renal syndrome (modified from [20])

Non-cell-based systems	Intermittent, extended and continuous dialysis techniques
	Hemoperfusion techniques
	Plasma exchange techniques
	Plasmapheresis
	Plasma filtration/adsorption
	Albumin dialysis • MARS • SPAD
	Prometheus
Cell-based systems (Bioartificial liver support systems)	Human hepatocytes (bioartificial liver support system)
	Porcine hepatocytes

1. *Single pass albumin dialysis*
  In SPAD, albumin is used as a component of the dialysate for more effective protein-bound toxin removal. The blood is placed in contact with a standard albumin-impermeable high-flux membrane and is dialyzed against an albumin-containing dialysate. Protein-bound molecules that are small enough to pass from the blood compartment through the membrane pores are dialyzed and then bound to albumin in the dialysate. SPAD provides a single pass of fresh albumin dialysate; this characteristic constitutes the major difference between SPAD and MARS [23, 24].
2. *Molecular adsorbent recirculating system*
  MARS uses a hemodialyzer in a primary circuit, which is connected to a secondary circuit composed of a standard hemodialyzer, an activated carbon adsorber and an anion exchanger. In the primary circuit, the patient’s blood is pumped into the MARS hemodialyzer and water-soluble substances diffuse through the dialysate solution. This membrane has a size selection threshold of less than 60 kDa, thus retaining albumin on the blood side; only the free fraction of toxins can cross the membrane in a manner similar to SPAD. The dialysate compartment of the MARS hemodialyzer is part of a secondary circuit, where a 20 % albumin solution circulates in a counter-current flow. Toxins can bind to the free albumin in the secondary circuit, while clearance of water-soluble substances occurs in a standard CRRT hemodialyzer. Hydrophobic albumin-bound toxins are then extractedby passage through activated charcoal and anion exchange columns, thus regenerating the albumin binding sites. The reconstituted albumin is then recirculated to maintain a transmembrane concentration gradient in the primary circuit hemodialyzer.
3. *Prometheus FPSA*
  The Prometheus system is based on FPSA combined with hemodialysis. The patient’s blood is pumped toward a specific albumin-permeable membrane with a size-selection threshold of 250 kDa. The albumin fraction of blood is selectively filtered and albumin-bound toxins can freely pass the membrane by convection. In a secondary circuit, the filtered albumin-rich plasma fraction is treated by two absorber columns: a neutral resin absorber and an anion exchanger for removal of negatively charged toxins. The purified albumin-rich plasma fraction is re-infused into the primary circuit where, in a second step, conventional hemodialysis is performed to eliminate water soluble molecules [25, 26].

##### Lung support

There is well-established evidence of interaction between lung and kidney functions and many critically ill patients may require concomitant extracorporeal kidney and lung support [27, 28]. In most cases, CRRT can be performed with the same vascular access used for extracorporeal lung support therapies, both for therapies requiring high blood flows (extracorporeal membrane oxygenation (ECMO) [29]) and, more recently, for therapies requiring low blood flows. ECMO is frequently performed in conjunction with CRRT and different circuit configurations can be used [27]. Conventional ECMO systems typically require blood flow rates substantially higher than those used in CRRT, although new therapies using lower blood flows may even be sufficient to achieve adequate extracorporeal oxygenation [30]. On the other hand, new lung support modalities utilizing blood flow rates similar to those applied in CRRT (and capable of being provided by CRRT machines) are sufficient to perform extracorporeal CO_2_ removal [31].

##### Blood purification in sepsis

In patients with hyper-inflammation (mainly during sepsis), extracorporeal blood purification therapies have the potential to modulate the host inflammatory response through the removal of inflammatory mediators and/or bacterial toxins.

1. *High-volume hemofiltration*
  Although not unequivocally defined in the medical literature, high-volume hemofiltration (HVHF; Fig. 2) is identified as continuous treatment with a convective target dose (prescribed) greater than 35 ml/kg/h [32, 33]. Continuous treatments with a dose greater than 45 ml/kg/h identify very high-volume hemofiltration (VHVHF) modalities. Intermittent procedures with brief, very high-volume treatments at 100 to 120 ml/kg/h for 4–8 h, followed by conventional CVVH, are identified as pulse HVHF [34].
  However, there is no evidence that HVHF, when compared with standard dose hemofiltration, leads to a reduction in mortality [35]. There is insufficient evidence to routinely recommend the use of HVHF in critically ill patients with severe sepsis and/or septic shock except as interventions being investigated in the setting of a randomized clinical trial.
2. *Continuous plasmafiltration coupled with adsorption*
  Continuous plasmafiltration coupled with adsorption (CPFA) is a blood purification therapy (Fig. 2) that combines the advantages of CRRT and continuous plasma filtration without requiring large amounts of plasma substitutes. In the first step of CPFA, a plasma filter separates plasma from the blood cellular component and the plasma filtrate is pumped through a sorbent. The purified plasma is then returned to the main circuit where blood is reconstituted and treated with standard CRRT modalities. There is no evidence that CPFA reduces mortality in patients with septic shock or that it positively affects other important clinical outcomes [36].
3. *Hemoperfusion*
  Continuous hemoperfusion involves placement of a sorbent cartridge in series with the filter (Fig. 2) in order to remove those toxins that are not removable by classic CRRT. The sorbent is placed in direct contact with blood and adsorbs solutes through hydrophobic interactions, ionic attraction, hydrogen bonds, and van der Waals interactions [37]. Hemoperfusion requires an extremely biocompatible sorbent coated with a surface that prevents platelet adhesion and clotting activation. The removal characteristics of hemoperfusion are dependent on the different types of sorbent used, with effective surface area playing an important role.
  Polymyxin (PMX)-hemoperfusion is a technique based on the use of a cartridge containing fibers coated with PMX B, an antibiotic with high affinity for lipopolysaccharide. The aim is to remove circulating endotoxin. Results from studies of PMX-hemoperfusion have been controversial. Nevertheless, the most recent results seem to suggest no improvement in organ failure in patients treated with PMX-hemoperfusion [38, 39].

## Conclusions

Application of technology at the bedside requires full knowledge of the basic principles and the operating mechanisms for every technique. When faced with a complex patient, practitioners can use a growing variety of extracorporeal treatment options. For patients with multiple organ failure, an increasingly rich panoply of options is being developed, including extracorporeal treatments for sepsis and for cardiac, pulmonary, and liver failure [40–44]. In this complex scenario, a multidisciplinary clinical care team composed of specialists from different disciplines and highly trained nurses is crucial to the success of the treatment. We suggest a framework for harmonization of terminology to reduce the errors and complications that can result from poor understanding and inadequate delivery of the prescribed therapies. Homogenized nomenclature is also important when reporting machine functions and clinical parameters to enable study comparisons and advance our understanding in this field, ultimately allowing for improvements in clinical practice and patient outcomes.

We trust that new publications, electronic medical records, and machine software will be designed and operated in compliance with the agreed terminology to enable consistent data collection and comparison.

## Abbreviations

AKI: Acute kidney injury
AVVH: Accelerated veno-venous hemofiltration
CPE: Continuous plasma exchange
CPFA: Continuous plasmafiltration coupled with adsorption
CRRT: Continuous renal replacement therapy
CVVH: Continuous veno-venous hemofiltration
CVVHD: Continuous veno-venous hemodialysis
CVVHDF: Continuous veno-venous hemodiafiltration
CVVHFD: Continuous veno-venous high-flux dialysis
ECMO: Extracorporeal membrane oxygenation
ED: Extended dialysis
EDD: Extended daily dialysis
EDDf: Extended daily dialysis with filtration
FPSA: Fractionated plasma separation and adsorption
HVHF: High-volume hemofiltration
ICU: Intensive care unit
IHD: Intermittent hemodialysis
IHDF: Intermittent hemodiafiltration
IHF: Intermittent hemofiltration
IHFD: Intermittent high-flux dialysis
MARS: Molecular adsorbent recirculating system
MOST: Multiple organ support therapy
PIRRT: Prolonged intermittent renal replacement therapy
PMX: Polymyxin
RRT: Renal replacement therapy
SCUF: Slow continuous ultrafiltration
SLED: Sustained low-efficiency dialysis
SLEDD: Slow low-efficiency extended daily dialysis
SPAD: Single pass albumin dialysis
TMP: Transmembrane pressure
TPE: Therapeutic plasma exchange
VHVHF: Very high-volume hemofiltration
